# To What Extent Do Disease-Modifying Anti-rheumatic Drugs Affect Bone Union in Trauma and Orthopaedic Patients?

**DOI:** 10.7759/cureus.95385

**Published:** 2025-10-25

**Authors:** Myat Pan, Johnathon Cann

**Affiliations:** 1 Trauma and Orthopaedics, Chelsea and Westminster Hospital NHS Foundation Trust, London, GBR; 2 Trauma and Orthopaedics, The Hillingdon Hospitals NHS Foundation Trust, London, GBR

**Keywords:** acute trauma care, bone healing, disease-modifying antirheumatic drugs, fracture union, rheumatoid arthritis, trauma

## Abstract

Patients with rheumatoid arthritis have an increased risk of postoperative complications in orthopaedic surgery because of the effect of the disease on bone as well as the immunomodulatory medications that can interfere with bone healing. Our literature review aims to synthesise current literature on disease-modifying anti-rheumatic drugs (DMARDs) available to aid decision-making in withholding or continuing these medications in the perioperative period. A literature search was conducted on Embase, PubMed, and MEDLINE. The initial search around foot and ankle osteotomies and DMARDs only yielded four original papers. After expanding our search to include trauma and elective procedures, the search yielded 80 papers. In total, nine papers were included for review after applying our inclusion and exclusion criteria. Methotrexate (MTX) appears to have a dose-dependent effect on bone healing with lower doses used in rheumatoid patients showing no adverse effect on bone healing. In spinal surgery, those who continued DMARDs had greater radiographic fusion outcomes and fewer disease flares compared to those who discontinued the drug. Biologic medications, however, showed adverse effects on bone healing and carried a higher rate of revision surgery. Our review has highlighted an important literature gap on the effects of DMARDs on bone union. The main finding from the review is that continuing conventional DMARDs in the perioperative period for joint surgery appears safe, but biologics should be held for at least two weeks or until there are signs of wound healing.

## Introduction and background

Introduction

An estimated 18 million people are living with rheumatoid arthritis (RA) in the world [1]. The disease comes with significant morbidity for patients, including the increased risk of fractures, compared to the general population, due to the chronic inflammation associated with RA, which can lead to reduced bone mineral density [2]. 

Presently, disease severity and progression have been helped by the rapid evolution of anti-rheumatic medications. These medications are broadly categorised into two main types: non-steroidal anti-inflammatory drugs (NSAIDs) and disease-modifying anti-rheumatic drugs (DMARDs) [3]. The DMARDs can be further classified into conventional and biologic. Conventional DMARDs include methotrexate (MTX), leflunomide, sulfasalazine, and hydroxychloroquine. Biologic DMARDs are newer, protein-based drugs that target specific components of the immune system to block the inflammatory response. Examples include tumor necrosis factor (TNF) inhibitors like infliximab and adalimumab. 

Patients with RA have an increased risk of postoperative complications from orthopaedic surgery because of the chronic impact of the disease on bone as well as the use of immunomodulatory medications that may interfere with bone repair [4]. Drugs with immunosuppressive action can lead to potential complications with both wound healing and bone union. This is particularly important in foot and ankle surgery, where corrective osteotomies are commonly performed and the risk of wound breakdown is high. Decisions to continue or suspend taking these medications need to be based on evidence, weighing the potential for postoperative complications versus potentiating a disease flare. 

There is an abundance of literature that highlights the adverse effects of NSAIDs on bone and fracture union, but there is little robust evidence surrounding the use of DMARDs in orthopaedic surgery [3,5]. Rheumatoid patients requiring surgery in trauma or elective settings will often be on one or more of these medications. Therefore, it is vital to understand the effect of DMARDs on postoperative outcomes to improve the recovery and rehabilitation of such a large patient cohort [6]. Current guidance published by the American College of Rheumatology has been formulated for elective hip and knee surgery with a focus on preventing wound complications, typically restarting DMARDs at the 14-day mark when the wound has healed. These guidelines do not consider the time to union of bone, which is typically six weeks [7]. This literature review aims to synthesise and evaluate the current evidence on the impact of DMARDs on bone healing. 

Methods 

A literature search was conducted on PubMed, Embase, and MEDLINE. An initial search was conducted looking at the effect of DMARDs or anti-rheumatic medications on bone union in foot and ankle osteotomies from dates of inception to March 2025. We used the keywords "DMARDs OR anti-rheumatic medications", "foot and ankle surgery OR osteotomy", AND "Bone healing OR bone union". It yielded only four original papers for review after removing duplicates, case reports, conference abstracts, and non-English-language materials. Due to the limited data in this field, we expanded our search and questions to look at the effects of DMARDs on bone union in elective and trauma patients. 

**Figure 1:**
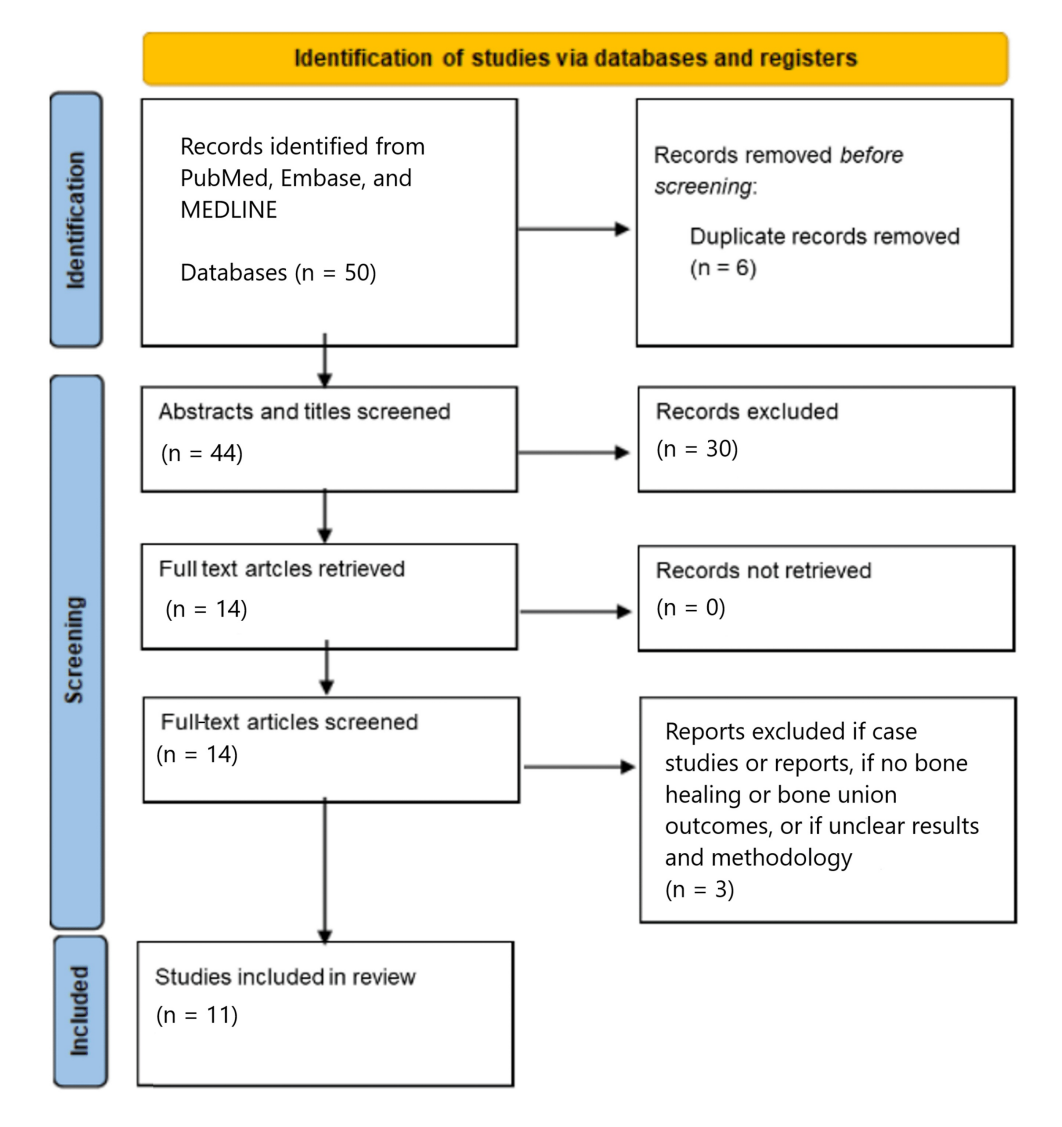
PRISMA flowchart for study selection PRISMA: Preferred Reporting Items for Systematic Reviews and Meta-Analyses

The keywords were subsequently refined to "rheumatic disease OR rheumatoid arthritis" AND "anti-rheumatic medication OR disease-modifying anti-rheumatic medications OR DMARDs" AND "fracture healing OR bony union OR mal-union or non-union". As there were still a limited number of original studies on this theme, we decided to include any study design apart from case studies. These were excluded due to their potential bias and limited generalisability. We also only included papers written in the English language and within the last 50 years. Although this is a qualitative review, we followed the Preferred Reporting Items for Systematic Reviews and Meta-Analyses (PRISMA) guidelines for article selection to ensure a clear and robust methodology [8]. We selected papers that looked specifically at either conventional or biologic DMARDs and their effect on bone healing, fracture union, or bone metabolism. The search resulted in 50 papers for review. After applying the above inclusion and exclusion criteria with two independent reviewers, a total of 11 papers were included for a narrative analysis. Figure 1 represents a flowchart of the article selection according to the PRISMA guidelines. 

## Review

Results 

Animal and In Vitro Studies 

Several preclinical studies have explored the effects of MTX and other DMARDs on bone healing. A key finding from this review is that the effect of DMARDs on bone appears to be dose-dependent. Satoh et al. compared bone formation in a fracture gap in rats between high- and low-dose MTX and control groups [9]. They showed that new bone formation did not differ significantly between the low-dose MTX and control groups. However, there was a marked reduction in bone formation in the high-dose MTX group, particularly periosteal bone formation de novo in the fracture gap site in the first week. The study showed no difference between the three groups for intramedullary bone formation or chondroid tissue formation. The authors acknowledge, however, that more data is available for the study of high-dose MTX on fracture healing in the literature, whereas less is known about the effects of low-dose MTX. Several other animal studies support the finding that high-dose MTX has a greater adverse effect on bone metabolism than low-dose MTX [10-12]. The lower doses are often used as a DMARD in inflammatory joint disease, but the disease process itself can interfere with bone turnover and remodelling. Therefore, it can be difficult to evaluate the effect of low-dose MTX in these patient populations. Satohet al.'s study, however, only analysed bone formation rather than bone strength or mineral density following the remodelling process [9]. 

In comparison, Pelker et al.'s rat model demonstrated that chemotherapy, including high-dose MTX, adversely altered rat bone biomechanics, reducing mechanical strength [10]. They tested fractured bone to failure in rapid torsion and measured the strength and stiffness of the healed bone over a course of 16 weeks. They found that MTX-treated bone led to a reduction in strength as well as stiffness compared to the control group. Histological examination suggests an abnormal remodelling process leading to reduced bone formation. This effect was even greater with higher doses, which supports the dose-dependent theory. 

Cavalcantiet al. observed similar effects in mandibular condylar fracture healing in rats, with high-dose MTX leading to delayed ossification, cartilage atrophy, and reduced bone formation [11]. Specifically, they discovered lower levels of alkaline phosphatase (ALP) in the high-dose cohort. ALP is a maker of osteogenesis, so this suggests suppressed bone formation. However, they only recorded data for 30 days. Similarly, Pountos et al.'s study also supports the view that high-dose MTX had more adverse effects on bone than low-dose MTX. Their systematic review analysed 70 papers of in vivo and in vitro studies on the effect of MTX on fracture healing [12]. They emphasised the limited and inconsistent data in this field. For instance, some in vitro studies included in their review showed that MTX negatively impacted mitochondrial activity, biochemical markers of osteogenesis, and cell turnover and differentiation which are crucial steps in bone healing or union, while other studies showed that MTX had no impact on these processes [3,13]. Notably, only three clinical studies were identified in their review, underscoring a major knowledge gap in translating preclinical findings into clinical practice [12]. 

Spinal Surgery Studies 

In clinical studies, the impact of DMARDs on bone union has been studied for patients undergoing elective spinal surgery [14-17]. A multicentre retrospective study by Elia et al. looked at bone fusion rates after craniovertebral junction surgery in 39 patients with RA on DMARDs [15]. Interestingly, they found that those who continued DMARDs showed higher radiographic fusion outcomes than those who discontinued their medication (92.8% vs. 75%; p=0.276). However, this finding was not statistically significant, likely due to their small study size. Moreover, four patients required reoperations: three from the discontinued group and one from the continued group. Two patients were readmitted for RA flare-ups, both of whom were from the discontinued group. The overall finding was that there were no statistically significant differences in surgical complications between the two groups, although they did not specifically assess the impact of DMARDs on bone healing. The authors believe that key factors affecting overall outcomes include comorbidities, disease severity, bone health, nutrition, and smoking rather than DMARD use alone [15]. 

Gaudiani et al. studied revision spinal surgery rates for patients on conventional DMARDs or TNF-alpha inhibitors compared to a control group not on either medication. The reoperation rate within one year was 19% for the TNF-alpha inhibitor group and 11% for the conventional DMARD group compared to 6% for the control group. The TNF-alpha group had a 3.1-fold increased risk compared to the control group (95% CI: 1.4-7.0), while the conventional DMARD group showed a 2.2-fold increase (95% CI: 0.96-5.3). The reasons for revision surgery were due to infection (40%) or other causes (60%), such as failure to fuse in the conventional DMARD group, while in the TNF-alpha inhibitor group, it was 47% for infection and 53% for other causes [16]. This implied that there is a higher rate of infection for the TNF-alpha inhibitor cohort. The authors concluded that TNF-alpha inhibitor use before spinal surgery led to significantly higher reoperation rates due to infection or mechanical failure. The use of other DMARDs still appeared to have a slightly higher-than-average risk of reoperation, but the statistics are less robust. In contrast to Elia et al., this study included 427 patients, so their results showed higher statistical power. 

Khanna et al.'s retrospective analysis on cervical spinal fusion surgery involved only 18 patients. They were either on MTX, prednisolone, or biologics for RA and craniovertebral joint disease [17]. The study found that MTX and biologics had no significant effect on spinal fusion, as only one patient did not achieve radiographic fusion. This was determined by postoperative computed tomography scans, but they did not specify the radiographic criteria for fusion and the duration of follow-up in these patients. In addition, this was an extremely small study size. A more recent 2022 systematic review by Mamaril-Davis et al. collated data from 294 DMARD-treated patients undergoing various spinal surgeries in six studies [14]. The data they examined suggested that patients who continued DMARDs had a higher risk overall of wound healing concerns and lower-than-average wound healing rates. The review focused mostly on wound complication rates rather than bone fusion outcomes, which was only described in one study and was statistically insignificant. 

Collectively, these findings suggest that conventional DMARDs, particularly MTX, appear relatively safe with respect to spinal fusion, while biologic DMARDs, especially TNF-alpha inhibitors, may increase perioperative risks, particularly infection. However, the evidence base remains limited, heterogeneous, and underpowered to make definitive conclusions. 

Foot and Ankle Surgery Studies 

Within foot and ankle surgery, Bibbo et al.'s review gave insight into the effect of anti-rheumatic agents on bone. They examined the records of 104 RA patients undergoing foot and ankle surgery on conventional DMARDs, steroids, and NSAIDs between 1980 and 2000 [18]. Although there was a notably high wound infection rate (32%), the authors could not prove any association with the use of DMARDs. It was thought that the higher risk of wound infection was due to the underlying disease and the complex nature of reconstructive surgery. These findings support the perioperative continuation of conventional DMARDs in foot and ankle surgery. 

Furthermore, a critical analysis review by Saunders et al. on the perioperative management of anti-rheumatic drugs in foot and ankle surgery also supports that conventional DMARDS are generally safe to use throughout the perioperative period, while biologics should be held typically before surgery [19]. They also acknowledged that more data is available on MTX than on any other conventional DMARDs, but there is reassuring safety data across multiple surgical settings. In comparison, the data for other conventional DMARDs in orthopaedic surgery are sparse, but the available evidence does not suggest significant harm. 

Guidelines 

Similarly, the data for biologic medications in general is scarce regarding bone healing. In 2017, the American College of Rheumatology and American Association of Hip and Knee Surgery (ACR/AAHKS) performed an extensive meta-analysis of the literature around the use of conventional and biologic DMARDs in orthopaedic surgery [7]. They focused particularly on the available literature on perioperative wound complications in patients with inflammatory arthritis undergoing elective hip and knee arthroplasty. In summary, they found no association between conventional DMARDs (including sulfasalazine, hydroxychloroquine, and leflunomide) and postoperative infections. On the other hand, they linked biologic DMARDs to increased rates of perioperative complications, including wound infection. As a result, the committee advised that conventional DMARDs can be continued throughout the perioperative period but recommended holding biologics for two weeks before surgery as there was an increased risk of wound-related complications. Notably, the guideline did not specifically address bone healing or union as an outcome, as the available literature focused almost exclusively on wound complications and infection. However, their recommendations have been widely adopted in orthopaedic practice and perioperative rheumatology protocols, and they remain the most influential evidence-based statement to date regarding DMARD management in surgery. Table 1 summarises the key findings of the literature reviewed. 

**Table 1:** Summary of evidence on DMARDs and bone union ACR: American College of Rheumatology; AAHKS: American Association of Hip and Knee Surgery; MTX: methotrexate; DMARDs: disease-modifying anti-rheumatic drugs; RA: rheumatoid arthritis; TNF-α: tumor necrosis factor alpha; pre-op: preoperative; SLE: systemic lupus erythematosus

Author (year)	Study type	Population/context	Conclusion
Goodman et al. (2022) [7]	Guidelines/meta-analysis	Guidance on DMARDs/biologics in elective hip and knee arthroplasty	Continue conventional DMARDs; hold biologics ~2 weeks pre-op; focus on wound outcomes
Satoh et al. (2011) [9]	Animal (rat model)	Fracture gap healing, high- vs. low-dose MTX vs. control	Low-dose MTX: no adverse effect. High-dose MTX: reduced periosteal bone formation
Pelker et al. (1985) [10]	Animal (rat model)	Chemotherapy effects on rat bone biomechanics	High-dose chemo-impaired biomechanics of bone
Cavalcanti et al. (2014) [11]	Animal (rat model)	MTX effect on mandibular condyle fracture healing	High-dose MTX delayed bone healing and cartilage repair
Pountos and Giannoudis (2017) [12]	Systematic review (in vivo and in vitro)	70 studies on MTX and fracture healing	High-dose MTX more adverse than low-dose MTX; evidence inconsistent; few clinical studies
Mamaril-Davis et al. (2022) [14]	Systematic review (6 studies, 294 patients, spine surgery)	DMARD-treated patients undergoing elective spinal surgery	DMARD continuation: higher wound concerns; fusion outcomes rarely reported
Elia et al. (2020) [15]	Multicentre retrospective (39 RA patients, spinal fusion)	RA patients on DMARDs undergoing craniovertebral surgery	Continued DMARDs: higher (non-significant) fusion rates, fewer flares
Gaudiani et al. (2021) [16]	Retrospective (427 spinal fusion patients)	Spinal fusion patients on TNF-α inhibitors or DMARDs vs. controls	TNF-α inhibitors: ↑ reoperation risk (OR 3.1); DMARDs: slight risk, not significant
Khanna et al. (2015) [17]	Retrospective (18 RA patients, craniovertebral fusion)	RA patients on MTX, steroids, and biologics undergoing craniovertebral fusion	No significant effect of MTX/biologics; 1 patient non-union; very small sample
Bibbo et al. (2003) [18]	Retrospective (104 RA patients, 725 foot/ankle procedures)	RA patients undergoing foot and ankle surgery, various DMARDs	32% complication rate; no association between DMARDs and wound/bone complications
Saunders et al. (2021) [19]	Critical analysis review	RA and SLE patients undergoing elective foot/ankle surgery	Conventional DMARDs generally safe; biologics should be held perioperatively

Discussion

Our narrative review has highlighted an important knowledge gap within the field of DMARDs and bone healing. The current literature are mostly animal studies suggesting that the effect of DMARDs on bone union is dose-dependent and may vary by drug class [9-12]. A consistent finding has been that high-dose MTX may have more adverse effects on bone than low-dose MTX, which is more commonly used as a DMARD in inflammatory joint disease. Importantly, preclinical studies focus more on high-dose MTX, creating an imbalance of evidence. They also primarily evaluate bone formation and cellular activity rather than long-term mechanical strength and bone mineral density, so it is difficult to assess the durability of bone repair with the use of these medications [3,13]. 

Human studies are even fewer, and the ones included in this review mostly have small study populations, have no randomisation, or are based in a single centre. Moreover, they are antiquated and often do not examine the latest anti-rheumatic drugs. For instance, an important study we included by Elia et al. included only 39 patients, which reduces the statistical power of the results [15]. All the clinical studies we have included so far are for elective procedures, exclusively in spinal surgery or foot and ankle surgery [14-19]. We have been unable to identify any literature beyond these operations or in a trauma setting. To our knowledge, there are currently no randomised controlled trials that study the effect of DMARDs on bone healing. Overall, the clinical literature further supports the preclinical studies that conventional DMARDs appear safe with respect to bone union, whereas biologics may be associated with an increased risk of postoperative infection and mechanical failure. 

Nevertheless, there are a greater number of publications available to consider for the effect of DMARDs on wound healing in orthopaedic surgery. This is of important consequence as surgical site infections, especially when involving the bone, can lead to impaired bone healing, causing mal-union or non-union [20]. Current evidence suggests MTX has no adverse effect on wound healing in orthopaedic surgery and can be safely continued pre- and postoperatively [11,12]. Biologics, on the other hand, are recommended to be held perioperatively due to the increased risk of surgical site infections and impaired wound healing. Current recommendation is to schedule the surgery at the end of the biologic dosing interval and restart medications roughly two weeks after the operation to allow surgical wound healing [11]. However, due to the scarcity of data for the effect of biologics on bone metabolism, it is difficult to determine whether two weeks is sufficient time to allow the bone to heal. Some hospital trusts have advised only restarting when most of the wound has healed [21]. Considering a wider evidence base, biologics have shown an increased risk of serious infection, so there is certainly a research gap to explore on how these medications impact patient outcomes generally in orthopaedic surgery [22]. 

Any decision to stop anti-rheumatic medications in the preoperative period should be a carefully weighed decision, with patients fully informed as to the risks and benefits of stopping such therapy. Patients on DMARDs tend to have more severe disease, and withholding them may result in disease flares, which can cause significant morbidity. Flares may lead to joint swelling, stiffness, pain, and increased cardiovascular risk [23]. This can ultimately impair rehabilitation following major surgery, which predisposes the patient to further postoperative complications such as venous thromboembolism, hospital-acquired infections, or reduced functional baseline from a prolonged hospital stay [24]. 

In fact, Grennan et al. found that those who discontinued MTX two weeks before and after surgery showed a higher rate of flare-ups than those who continued their medication. Patients who continued MTX before surgery had even fewer postoperative complications than the control group not on any MTX [25]. The 2017 American College of Rheumatology study also concluded that continuing glucocorticoids and DMARDs perioperatively for hip and knee arthroplasty resulted in better function, a greater range of motion, and improved postoperative pain [7]. Therefore, we advise that the decisions around anti-rheumatic medications in patients undergoing orthopaedic surgery should be determined on an individual basis, with consideration given to their disease severity, functional baseline, and risk factors for poor bone healing, as we currently do not have enough evidence to suggest that they should be held. Further research should prioritise prospective or randomised controlled trials to assess radiographic and biomechanical union in orthopaedic patients treated with DMARDs. Animal studies may also be helpful in exploring the long-term effect of DMARDs on bone quality and strength as well as including biologics in trials. 

A key limitation across the field is the paucity of well-designed clinical studies directly measuring bone healing or union rates in patients on DMARDs. Most of the available evidence is drawn from small retrospective series, with limited statistical power and heterogeneity in surgical techniques, dosing regimens, and outcome measures. Moreover, confounding from underlying disease severity, systemic inflammation, nutrition, smoking, and comorbidities complicates the isolation of DMARD effect [14-17]. Therefore, the results of our review are limited by these factors and should be interpreted with caution. Moreover, we used specific terminology to capture the effect of anti-rheumatic medications on bone healing, so we may have missed articles that contain this information, which did not include our keywords. The topic is focused, and we note there may be an element of selection bias as a result of the search strategy. To note, preliminary findings for this review were published in a pre-print server [26]. 

## Conclusions

The effect of DMARDs on bone union remains largely unstudied, especially considering human studies and large randomised controlled trials. Our literature review has identified that MTX may be safe to continue before orthopaedic surgery as it does not appear to affect bone union at low doses that are used in RA. Biologic DMARDs, on the other hand, should be withheld as some evidence suggests they can cause an increased risk of infection or wound breakdown. However, the decision to withhold any anti-rheumatic medication should be weighed against the risk of flare-ups. Given that this is a growing patient population, it is important to further understand the clinical impact of DMARDs on bone so that evidence-based guidance can be given. Until then, we advise a multidisciplinary approach in determining which anti-rheumatic medications to withhold before any orthopaedic surgery. 
